# Presenteeism and Traffic Accident Among Taxi Drivers: A Prospective Cohort Study in Japan

**DOI:** 10.1016/j.shaw.2024.04.002

**Published:** 2024-04-16

**Authors:** Makoto Okawara, Kei Tokutsu, Keiki Hirashima, Tomohiro Ishimaru, Yoshihisa Fujino

**Affiliations:** 1 Department of Environmental Epidemiology, Institute of Industrial Ecological Sciences, University of Occupational and Environmental Health, Japan; 2 Department of Preventive Medicine and Community Health, School of Medicine, University of Occupational and Environmental Health, Japan; 3 Department of Medical Humanities, School of Medicine, University of Occupational and Environmental Health, Japan

**Keywords:** Cohort studies, Japan, Occupational groups, Traffic accidents

## Abstract

**Background:**

Traffic accidents involving professional drivers have serious societal repercussions. Unique occupational stressors and health risks exacerbate the likelihood of traffic accidents among professional drivers. This study explores the association between presenteeism—impaired work performance due to working while unwell—and traffic accident risk among professional taxi drivers in Japan.

**Methods:**

A prospective cohort study was conducted from June 2022 to February 2023, involving taxi drivers from a single company in Fukuoka Prefecture, Japan. Presenteeism was assessed using the Work Functioning Impairment Scale (WFun). Primary outcome involved the number of self-reported minor traffic accidents. The incidence rate ratio (IRR) of minor traffic accident occurrences was estimated using a Poisson regression analysis, adjusted for confounders including sex, age, and driving experience.

**Results:**

Of 838 targeted drivers, 435 were included in the analysis. Higher baseline work functioning impairment was associated with a significant trend of increasing IRR of minor traffic accidents (*p* for trend = 0.045). A dose-response relationship was seen between the degree of presenteeism and incidence rate of minor traffic accidents.

**Conclusion:**

Higher levels of presenteeism were associated with an increased risk of traffic accidents among taxi drivers. The findings underscore the need for socio-economic support and prioritized health management to mitigate traffic accident risk among professional drivers. This study highlights the importance of managing non-critical health issues alongside serious health conditions for safer driving practices among professional drivers in Japan.

## 1 Introduction

Traffic accidents involving professional drivers of commercial vehicles have a significant social impact. Traffic accidents caused by professional drivers result in greater social accountability for the driver and their employer than those caused by the general public. Transporting multiple passengers, large cargo, and hazardous materials increases health risks in traffic accidents involving professional drivers. Compared to the general public, professional drivers often face longer driving hours, extended work periods, irregular routes, and time pressures [[1], [2], [3], [4], [5]]. These factors heighten traffic accident risk for professional drivers.

Health management in professional drivers affects traffic accident risk. Seizures, strokes, cardiovascular events, and sudden death can cause a complete loss of consciousness, making driving impossible and resulting in a serious traffic accident. Cardiovascular disorders increase traffic accident risk [6]. Aside from complete loss of consciousness, common health conditions such as fatigue and drowsiness are also related to traffic accidents. Driving requires advanced functions to oversee the entire activity, maintain a relationship with the surrounding environment using cognitive abilities, and convert information into actions like braking, accelerating, and steering [7]. Health conditions like fatigue and drowsiness impair cognitive functions, increasing traffic accident risk. In many countries, fatigue and drowsiness contribute significantly to traffic accident [[8], [9], [10]].

Many professional drivers experience poor health conditions. Professional drivers are reported to be at elevated risk for conditions like hypertension, obesity, and diabetes [6,11,12]. Professional drivers who are often confined to their vehicle report back pain, low physical activity, high smoking rates, and irregular sleep patterns. Sudden driving dangers heighten stress, activate the sympathetic nervous system, and affect cardiovascular dynamics [13]. The complexity of these health risks makes assessing the relationship between professional drivers’ individual health condition and traffic accidents challenging. The link between such a wide range of poor health conditions and traffic accident risk due to health-related factors is unclear.

Professional drivers with poor health conditions exhibit presenteeism, a condition of impaired work performance due to working while unwell [14]. Previous studies link presenteeism to workplace errors [15]. While past research has emphasized the relationship between specific health conditions of professional drivers and traffic accidents, we interpret professional drivers’ complex health risks through the lens of presenteeism. Although presenteeism may relate to professional drivers’ traffic accidents, this relationship remains unclear.

We have hypothesized that professional drivers experiencing presenteeism are at elevated risk of traffic accidents. Here, we investigated this possible association between presenteeism in professional drivers and traffic accidents at work.

## 2 Methods

### 2.1 Subject and settings

We conducted a prospective cohort study using a baseline survey in June 2022 and a follow-up survey in February 2023. Taxi drivers employed at a single company in Japan were targeted. This company, located in a specific region of Fukuoka Prefecture, Japan, operates 17 branches and employs 838 taxi drivers. We recruited from all branches and employees of this company for this voluntary survey. Drivers were recruited through branch managers and requested to complete the survey forms. Both baseline and follow-up surveys were paper-based, self-administered questionnaires. This study was approved by the Ethics Committee of the University of Occupational and Environmental Health, Japan (Approval Number: R4-014).

### 2.2 Assessment of presenteeism and other covariates

In the baseline survey, we gathered participant characteristics and presenteeism. Presenteeism was assessed using the Work Functioning Impairment Scale (WFun). WFun, developed using the Rasch model, measures work functioning impairment and has been validated in accordance with COSMIN [16]. WFun consists of seven questions with scores between 7 and 35 points. Its reliability and responsiveness have been validated against benchmarks like the Stanford presenteeism scale, 8-item Short Form Health Survey, and Work Ability Index [16,17]. Its responsiveness has also been validated against several indicators reflecting the severity of pain associated with depression and musculoskeletal disorders [18,19]. Based on prior studies correlated with evaluations by occupational health nurses, we categorized WFun scores of 7–13 as Low, 14–20 as Middle, and 21 and above as High [20].

Potential confounders were evaluated from among baseline information on sex, age, driving experience (number of years with current company), and weekly driving hours.

### 2.3 Measurement of traffic accidents at follow-up

The primary outcome was the number of minor traffic accidents, defined as self-reported minor traffic accidents such as scrapes and collisions occurring over the past three months, even those not reported to the company. We identified minor traffic accidents by asking the following questions: ‘How many minor traffic accidents, such as scrapes or slight hits, have you experienced in the last three months, including those that you did not report to the company?’

### 2.4 Statistics

We estimated the incidence rate ratio of minor traffic accident occurrences using a Poisson regression analysis with robust standard errors, using actual number of minor traffic accidents as outcome variables and weekly driving hours as an offset term. The multivariate model was adjusted by sex, age, and driving experience. All analyses were performed using Stata (Stata Statistical Software: Release 17.0; StataCorp LLC, TX), with a *p* < 0.05 indicating statistical significance.

## 3 Results

**Fig. 1:**
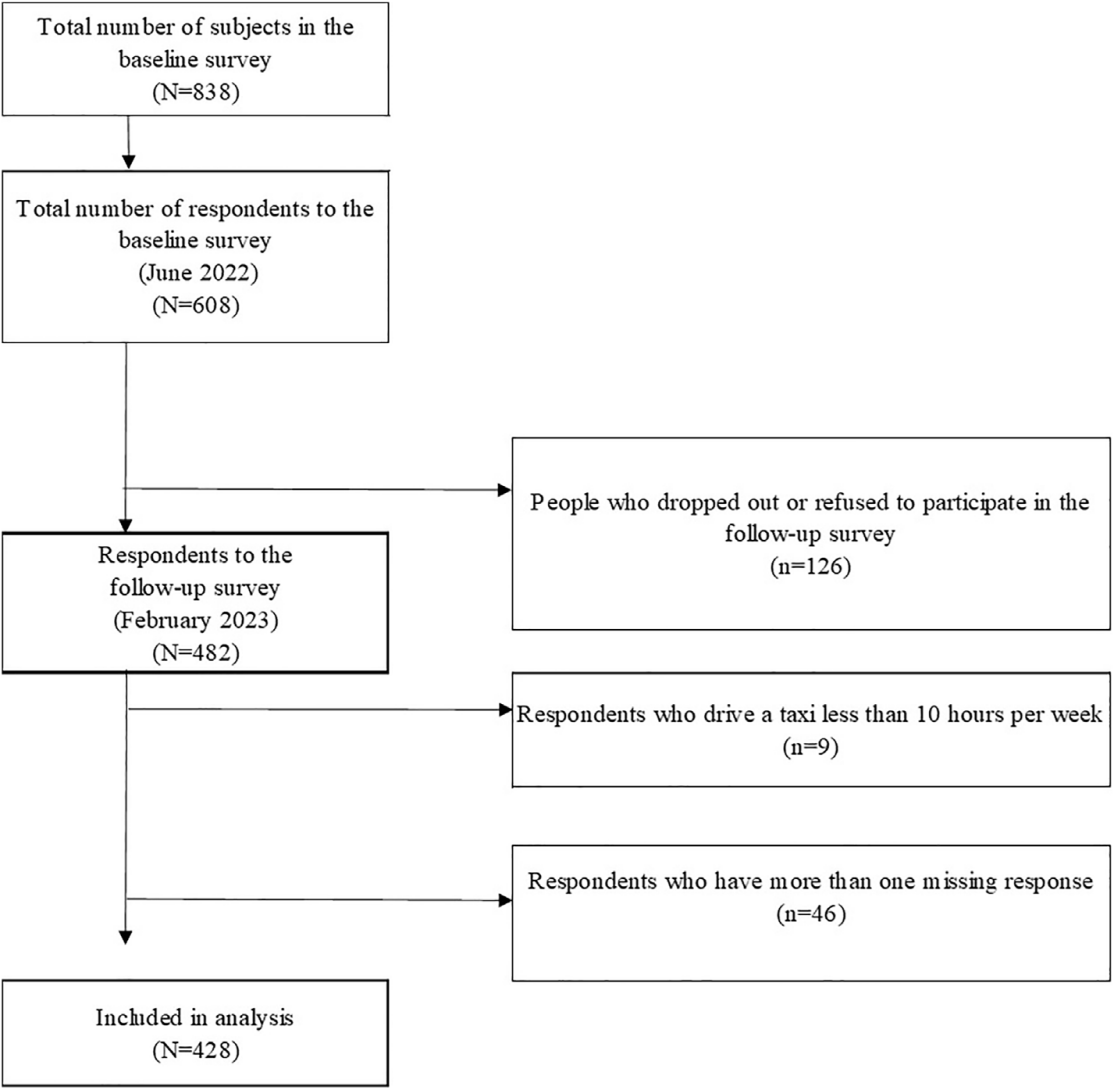
Flow chart of participants in the study.

Fig. 1 shows the participant flowchart. Of the 838 drivers targeted in the baseline survey, 608 (73%) participated, and 482 (79%) took part in the follow-up. We excluded participants driving under 10 hours weekly (*n* = 9; 1.9%) and those with missing essential data (*n* = 46; 9.5%) (Supplementary Table). We included 428 individuals in the analysis.

Table 1 shows the participants’ baseline characteristics. 93% of participants were male. Median age was 67 years, and 25% were aged under 60 years, indicating the inclusion of many older drivers.

**Table 1:** Participant demographic and sociological characteristics

Variable	Total		Work functioning impairment scale					
			Low (7–13)		Middle (14–20)		High (21–35)	
	*n* = 428		*n* = 343		*n* = 72		*n* = 13	
	*n*	%	*n*	%	*n*	%	*n*	%
Age, years, median (interquartile range; IQR)	67 (60–72)		67 (60–72)		66 (61–71)		59 (57–67)	
20–59	107	25.0	85	24.8	15	20.8	7	53.8
60–66	96	22.4	72	21.0	23	30.6	2	15.4
67–71	104	24.3	83	24.2	19	26.4	2	15.4
72–79	121	28.3	103	30.0	16	22.2	2	15.4
Sex, men	399	93.2	320	93.3	69	94.4	11	84.6
Hours of driving taxi per week, median (IQR)	30 (20–40)		30 (20–42)		30 (20–36)		25 (22–48)	
Experience of taxi driver, years, median (IQR)	9 (4–17)		9 (4–17)		9 (3–18)		12 (5–18)	
Score of Work functioning impairment scale at baseline, median (IQR)	9 (7–12)		8 (7–10)		15 (14–17)		22 (21–25)	

The actual number of minor traffic accidents by baseline work functioning impairment are as follows: Low impairment - 84% had 0 incidents, 15% had 1, 0.6% had 2, and 0.3% had 3; Middle impairment - 83% had 0 incidents and 17% had 1; High impairment - 77% had 0 incidents, 15% had 1, and 8% had 2.

Table 2 shows the link between baseline work functioning impairment and minor traffic accidents in the three months preceding the follow-up survey. Groups with higher baseline work functioning impairment had a greater proportion of one or more minor traffic accidents. Poisson regression analysis revealed rising incidence rate ratios of minor traffic accidents in groups with more baseline work impairment. A trend test revealed a significant increase in the incidence rate ratio with increasing WFun score (*p* for trend = 0.045).

**Table 2:** Association between driver’s work functioning impairment and experience of minor traffic accidents

Outcome variables	Explanatory variables		*n* = 428	More than one minor traffic accident	Univariate				Multivariate∗			
			%	%	Incidence rate ratio	95% confidence interval		*p*	Incidence rate ratio	95% confidence interval		*p*
Experience of minor traffic accidents in the past three months	Work functioning impairment	Low	80	16	reference			0.074†	reference			0.045†
		Middle	17	17	1.04	0.58	1.87	0.884	1.04	0.57	1.88	0.899
		High	3	23	1.68	0.50	5.67	0.404	1.90	0.54	6.71	0.319

Using weekly driving hours as an offset term.

∗ Adjusted for sex, age, experience of taxi driving.

† *p* for trend.

## 4 Discussion

We studied the association between presenteeism and traffic accidents in a prospective cohort of taxi drivers. We observed a dose-response relationship: drivers with higher work functioning impairment scores showed increased incidence rates of minor traffic accidents. Few studies have explored the link between presenteeism and actual work output indicators despite significant interest [21,22]. Our research has implications in today’s industrial structure, where measuring direct work outputs is challenging.

Health conditions linked to presenteeism may increase traffic accident risk by affecting driving capabilities. Presenteeism arises from fatigue, drowsiness, pain, mental health issues, and more. These symptoms impair the function of concentration and judgment, causing mistakes. A previous study noted a link between presenteeism and near-misses among emergency personnel [15]. Delays in reaction time and impaired decision-making due to fatigue or drowsiness and decreased judgment can lead to reduced attention and increased traffic accident risk [[23], [24], [25], [26], [27], [28], [29], [30], [31]]. Sleep deprivation affects performance in a similar way to alcohol consumption [[32], [33], [34]]. Patients with sleep-disordered breathing often experience occupational accidents [35]. Chronic pain may increase traffic accident risk [36]. Individuals with sleep disorders, pain, and depression have been found to score highly on the WFun scale used in this study [18,19,37].

Socioeconomic status and working conditions might exacerbate this issue in professional drivers. Japanese taxi drivers earn about 30% less than the average industry wage as of 2022 [38,39]. Many receive commission-based pay, relying on picking up and driving passengers to earn sufficient income [40]. Drivers carrying more passengers are often valued higher, incentivizing them to work more frequently and longer hours. In these circumstances, drivers might work while unwell to sustain their income. Indeed, 30% of taxi drivers in a Japanese region admitted to driving while unwell, of whom 58% cited potential income loss as the reason [41]. A phenomenon termed ‘Sickness Presenteeism’ - defined as risk behavior of working while unwell [[42], [43], [44]] is a frequent and well known occurrence in individuals facing job insecurity or lack of workplace support such as adequate sick leave policies [45]. Higher work commitment among the Japanese may also contribute to presenteeism [46].

We may have overlooked the impact of non-critical health problems on traffic accident risk despite their importance. Two reasons explain this. First, attention to traffic accidents involving professional drivers has mainly targeted cerebrovascular and cardiac diseases causing sudden unconsciousness or death. However, in the past decade in Japan, only 30% of professional driver traffic accidents stemmed from brain, heart, or major vascular diseases [47]. Additionally, unlike cerebral vascular disorders, non-critical health problems obscure the cause of traffic accidents, possibly leading to underreporting. Second, minor traffic accidents were often overlooked while attention was focused on severe accidents. Traffic accidents from non-critical health problems often result in drivers staying conscious, leading to more minor or self-inflicted traffic accidents, unlike those following a complete loss of consciousness. Since drivers don’t always report such traffic accidents to police or companies, their actual prevalence is unknown. Nevertheless, continuing to drive under such conditions could result in the exacerbation of minor traffic accidents to severe ones.

Managing non-critical health problems is vital in preventing traffic accidents among professional drivers. Japan has focused on preventing traffic accidents in professional drivers by addressing diseases leading to sudden death or unconsciousness. Japan has instituted mandatory health examinations and recommended screening for vascular diseases and sleep disorders. In contrast; however, routine management of non-critical health problems has largely been underweighted. In Japan, operations managers require professional drivers to undergo mandatory pre-service roll calls and basic health checks. The specifics of these basic health checks are not well-defined. Drivers more frequently cause minor traffic accidents than severe ones. Managing non-critical health problems is critical for traffic accident prevention. Our present findings highlight the potential utility of assessing professional drivers’ health management in light of presenteeism.

This study has several limitations. First, concern has been expressed on the reliance on self-reported traffic accident experiences. Additionally, we were unable to ascertain the details of minor traffic accidents. However, minor traffic accidents often go unrecorded unless severe, so reliance on individual accounts is unavoidable. Second, we did not consider changes in health status from baseline. The actual association may be stronger than reported because of potential dilution of the relationship if health status changed post-baseline. Third, our understanding of confounding environmental factors linked to traffic accidents is insufficient. We did not consider factors like driving times, traffic volume differences, or regions, and the impact of these factors remains uncertain. This study focused on taxi drivers in a specific area with relatively consistent working hours and driving zones. Finally, we did not consider individual factors like specific illnesses of drivers, medication use, or external work stressors. However, given that presenteeism results from these factors, the absence of this information is not a significant issue. Adjusting for these factors would be over-adjustment.

In conclusion, our study shows that higher presenteeism among Japanese taxi drivers is associated with heightened traffic accident risk. To prevent traffic accidents, socio-economic support for professional drivers should be strengthened and their health management should be prioritized.

## Funding

This study was supported and partly funded by a research grant from the 10.13039/100016239University of Occupational and Environmental Health, Japan (UOEH Research Grant for Promotion of Occupational Health; no grant number). No funder was involved in the study design, collection, analysis, interpretation of data, the writing of this article, or the decision to submit it for publication.

## Conflicts of interest

Dr. Fujino holds the copyright to WFun with royalties paid from Sompo Health Support Inc., outside of this work. The other authors declare no conflicts of interest associated with this manuscript.
