# Supplementary material for: Presenteeism and Traffic Accident Among Taxi Drivers: A Prospective Cohort Study in Japan
Source: Saf Health Work. 2024 Apr 16;15(2):208–12. doi: 10.1016/j.shaw.2024.04.002 (PMC11255960; doi:10.1016/j.shaw.2024.04.002)
Supplement: Multimedia component 1 [file mmc1.docx]

| Supplementary table. Distribution of excluded data | |  |  |
| --- | --- | --- | --- |
|  |  | N = 482 | |
|  |  | Missing data | |
| Variable | | n | % |
| Respondents who drive a taxi less than 10 hours per week | | 9 | 1.9 |
| Any of following variables missing | | 46 | 9.5 |
|  | Hours of driving taxi per week | 28 | 5.8 |
|  | Experience of taxi driving | 1 | 0.2 |
|  | Work functioning impairment scale | 13 | 2.7 |
|  | Experience of minor traffic accidents in the past three months | 7 | 1.5 |
